# The effectiveness and safety of nicorandil in the treatment of patients with microvascular angina: A protocol for systematic review and meta-analysis

**DOI:** 10.1097/MD.0000000000023888

**Published:** 2021-01-15

**Authors:** Ye Zhang, Xiaojuan Wang, Ruijuan Liu, Qingsheng Li, Wei Tian, Hong Lei, Conghong Shi

**Affiliations:** a Nursing Teaching and Research Section of Medical Department, Hetao College, Yunzhong Street, Shuanghe Town, Linhe District, Bayannur; b Parasitology Teaching and Research Section of School of Basic Medical Sciences, Inner Mongolia Medical University, Jinshan Development Zone; c Department of Pharmacy, the Affiliated Hospital of Inner Mongolia Medical University, NO.1 Tongdao North Road, Huimin District, Hohhot 010050; d Department of Pharmacy, Bayannur Hospital, NO.98 Wulanbuhe Road, Linhe District, Bayannur; e Department of Pharmacy, Inner Mongolia People's Hospital, NO.20 Zhaowuda Road, Saihan District, Hohhot; f Department of Cardiovascular Medicine, the Fourth Hospital of Baotou, NO.29 Aogeng Road, Qingshan District, Baotou, Inner Mongolia Autonomous Region, PR of China.

**Keywords:** nicorandil, microvascular angina, X syndrome, coronary microvascular dysfunction, protocol, systematic review and meta-analysis

## Abstract

**Background:**

Microvascular angina has become a clinical and frequent cardiovascular disease in recent years, which is complicated and there is no clear treatment. Today, Western medicine still deals with microvascular angina with standardized treatment based on the stable angina. Firstly, it is to control the risk factors of atherosclerosis, and the second is to reduce the oxygen consumption of the patient's heart muscle. In the previous randomized controlled clinical trials, it has shown that nicorandil can improve the symptoms of angina for the treatment of microvascular angina, but there is a lack of high-quality randomized controlled trials on the clinical effectiveness and safety of nicorandil in the treatment of microvascular angina, and the lack of evaluation of its effectiveness and safety. Therefore, this paper aims to understand whether nicorandil can further improve the prognosis of patients with microvascular angina and the safety of the drug through the method of systematic evaluation.

**Methods:**

Retrieval of relevant network electronic databases by computer: SinoMed, CNKI, WanFang Data, VIP, PubMed, EMbase and The Cochrane Library, the retrieval time is from the establishment of each database to December 2017, to collect randomized controlled studies of nicorandil in the treatment of microvascular angina. At the same time, it is supplemented by manual search of the included literature references, as far as possible to increase the included literature imformation. Two researchers independently browse the topics and abstracts, and select, find, read the full text of the relevant literature, and screen the literature according to the criteria for inclusion and exclusion established in advance, then extract the data, and cross-check, and resolve the differences through multi-person discussion. Data analysis of collected information is performed by using RevMan 5.3 software.

**Results:**

The data of the included literature are statistically analyzed by meta-analysis, and the key outcome indicators are used to determine whether nicorandil can further improve the prognosis of patients with microvascular angina and the safety of the drug.

**Conclusion:**

Through the method of evidence-based medicine, this study finds the existing problems and defects in the current research, which will provide high-quality evidence-based medical evidence for nicorandil's treatment of microvascular angina, and it help the clinical treatment and further research.

**OSF registration number:**

DOI 10.17605/OSF.IO/DSQG9.

## 1 Introduction

Coronary microvascular dysfunction is divided into 4 types: the first is the type of non-combined obstructive coronary disease and cardiomyopathy, which mainly manifestes as microvascular angina (MVA); the second is the type of combined cardiomyopathy, which mainly includes hypertrophic cardiomyopathy, dilated cardiomyopathy, myocardial amyloidosis, myocarditis, aortic stenosis, anderson syndrome, and so on; the third type is the type of combined obstructive coronary disease, which includes stable angina, acute coronary syndrome, and the fourth is medical coronary injury, which includes coronary intervention therapy, coronary bypass transplantation.^[1–4]^ Angina is the main clinical manifestation of ischemic heart disease, which dues to the imbalance between heart oxygen supply and oxygen demand, resulting in the accumulation of metabolites, and causes angina symptoms, so that patients with an increased risk of cardiovascular events, and the life, health and quality of life are threatened.^[5]^

MVA is also known as Cardiac syndrome X, and it is a type of coronary microvascular dysfunction, which originally proposed by Cannon and Epstein in 1988 to describe patients with normal coronary angiology and a group of clinical syndromes of myocardial ischemic symptoms caused by coronary microvascular lesions.^[6]^ Because the creating agent can only enter the blood vessels with a diameter ≥0.5 mm, it can only be diagnosed as microvascular angina by clinically having typical labor-type angina symptoms or moving load tests to detect ischemic ST segment movement down.^[7,8]^ The pathogenesis of the disease is not clear, its pathogenesis may involve coronary structure and dysfunction, endotrical insanity, inflammatory response, estrogen deficiency, mental-neuro-endocrine disorders and other factors, and in different individuals, the pathogenesis is not the same.^[9,10]^ The treatment strategies of MVA patients are mainly to improve myocardial ischemia, dilate coronary microvessels, reduce myocardial oxygen consumption, and increase the stability of endothelial function. The main management goals of MVA patients are:

(1) Improve or eliminate myocardial ischemia and correct the cause;
(2) Improve the quality of life of patients;
(3) Manage changeable risk factors. As there is no clear diagnostic criteria for MVA, according to the *2016 Guidelines for the Rational Use of Coronary Heart Diseases*, it is recommended to use β-blockers, nitrates, calcium antagonists, angiotensin converting enzyme inhibitors, and nicorandil and other treatments.^[11]^

Studies have shown that nicorandil can achieve the goal of improving the symptoms of MVA patients by inhibiting inflammatory factors and improving the function of vascular endothelial, and the effect is more significant.^[12]^ Nicorandil is a new type of vasodilator, and it not only a nitrate compound, but also an adenosine triphosphate (ATP)-sensitive potassium channel opener. Nicorandil can convert nitric oxide (NO) through the participation of enzymes and relax vascular smooth muscle. It effectively expands the coronary arteries and capillaries in the patient's body, and it can stimulate the body's ATP-sensitive potassium channels by inducing guanylate cyclase, reduce Ca^2+^ influx, increase intracellular K^+^ outflow, reduce intracellular Ca^2+^ levels, and promote cells hyperpolarization, improve vascular endothelial function, significantly expands microvessels with a diameter of 100-200 μm, increase blood flow, and achieve the purpose of treating microvascular angina.^[13]^

However, there is currently a lack of high-quality randomized controlled trials on the clinical effectiveness and safety of nicorandil in the treatment of microvascular angina. Therefore, this study applies the international evidence-based medicine general method of systematic evaluation to systematically review the clinical research of angina pectoris. The purpose is to provide a theoretical basis for the drug treatment of microvascular angina.

## 2 Research purposes

The clinical research of nicorandil in the treatment of microvascular angina at home and abroad is systematically evaluated with the method of systematic evaluation of international evidence-based medicine. According to the principle of systematic evaluation of population, intervention, comparison, outcomes and study, the retrieval strategy was formulated. The subjects are patients with clinical diagnosis of microvascular angina. In this paper, through the method of systematic evaluation, the purpose of this study is to understand whether nicorandil can further improve the prognosis of such patients and the safety of the drug, find out the existing problems and defects in the current research, and provide ideas for the next research.

## 3 Methods

### 3.1 Research registration information and ethical approval

#### 3.1.1 Research registration information

This research has been registered on the OSF. Registration number was DOI 10.17605/OSF.IO/DSQG9. (https://osf.io/dsqg9, date registered: November 2, 2020).

#### 3.1.2 Ethics and dissemination

The data in this research comes from published articles in professional literature databases and Internet resources, so ethical review and approval are not required.

### 3.2 Search strategy

In this study, we followed the guidelines for systematic review and meta-analysis. We searched the foreign language database by computer: PubMed (https://www.ncbi.nlm.nih.gov/pubmed/), Web of Science (www.webofknowledge.com/), Embase (https://www.embase.com/), the Cochrane Library (https://www.cochranelibrary.com/). Chinese database: CNKI (https://www.cnki.net/), WanFang Data (http://www.wanfangdata.com.cn/), SinoMed (http://www.sinomed.ac.cn/). The system of literature retrieval was carried out, and the search databases were published from the database to October 1, 2020. The randomized controlled study of nicorandil in the treatment of microvascular angina. The search language is limited to Chinese and English. Chinese literature search terms: nicorandil, microvascular angina, cardiac X syndrome, etc. The steps of English literature retrieval are as follows: search subject words (the key words designated by the official database) + free words (referring to the key words formulated by ourselves). Taking PubMed database as an example (Table 1).

**Table 1:** Retrieval strategy of PubMed database by MeSH terms and keywords.

**Pubmed**	
1	Nicorandil[Mesh]
2	Microvascular Angina[Mesh]
3	Microcirculation[Mesh]
4	Coronary Occlusion[MeSH Terms]
5	Coronary Circulation[Mesh]
6	(nicorandil[Title/Abstract]) or (2-nicotinamidoethyl nitrate[Title/Abstract]) or (2 nicotinamidoethyl nitrate[Title/Abstract]) or (nitrate, 2-nicotinamidoethyl[Title/Abstract]) or (2-nicotinamidethyl nitrate[Title/Abstract]) or (2 nicotinamidethyl nitrate[Title/Abstract]) or (nitrate, 2-nicotinamidethyl[Title/Abstract]) or (SG-75[Title/Abstract]) or (SG 75[Title/Abstract]) or (SG75[Title/Abstract]) or (Ikorel[Title/Abstract]) or (adancor[Title/Abstract]) or (dancor[Title/Abstract])
7	(microvascular angina[Title/Abstract]) or (coronary microvascular dysfianction[Title/Abstract]) or (angina, microvascular[Title/Abstract]) or (X syndrome, angina[Title/Abstract]) or (angina X syndrome[Title/Abstract]) or (angina X syndromes[Title/Abstract]) or (syndrome, angina X[Title/Abstract]) or (syndrome X, cardiac[Title/Abstract]) or (syndrome X, angina[Title/Abstract]) or (angina syndrome X[Title/Abstract]) or (angina syndrome Xs[Title/Abstract]) or (syndrome Xs, angina[Title/Abstract]) or (angina pectoris with normal coronary arteriogram[Title/Abstract]) or (cardiac syndrome X[Title/Abstract])
8	(microcirculation[Title/Abstract]) or (coronary microcirculatory disorder[Title/Abstract])
9	(coronary occlusion[Title/Abstract]) or (coronary occlusions[Title/Abstract]) or (occlusion, coronary[Title/Abstract]) or (occlusions, coronary[Title/Abstract])
10	(coronary circulation[Title/Abstract]) or (circulation, coronary[Title/Abstract])
11	1 or 6
12	2 or 3 or 4 or 5 or 7 or 8 or 9 or 10
13	11 and 12

### 3.3 Eligibility criteria

#### 3.3.1 Types of researches

The selected studies are all randomized controlled studies that reported nicorandil as an anti-microvascular angina.

#### 3.3.2 Types of participant

For specific groups of people with microvascular angina, and without combining obstructive coronary disease and cardiomyopathy, the subjects should be older or equal to 18 years of age, regardless of gender.

#### 3.3.3 Inclusion criteria

The diagnostic criteria of “microvascular angina” are:

(1) Typical exertional angina symptoms, or atypical chest pain episodes, and different durations;
(2) Definite evidence of myocardial ischemia: (I)an electrocardial motion test or dynamic electrocardial chart has isoemia changes in the heart muscle;(II)myocardial perfusion imaging shows the evidence of myocardial ischemia;(III)magnetic resonance scan has changes in myocardial perfusion defect;
(3) Coronary antomy shows non-obstructive coronary artery stenosis, coronary diameter stenosis < 50% and coronary blood flow reserve fraction (fractional flow reserve) > 0. 8;
(4) Coronary flow reserve decreases or coronary microcirculation spasm. MVA can be diagnosed if the above 4 conditions are met, and highly suspected MVA cases can be diagnosed if any 1 of (1), (3) plus (2) or (4) is met.^[14]^

#### 3.3.4 Types of interventions and comparisons

The experimental group is given nicorandil + basic treatment (same as the control group), and the route of administration could be oral or intravenous injection, except for intracoronary injection. The course of medication is not limited. The control group is given conventional treatment: use drugs to reduce platelet aggregation, control the content of low-density lipoprotein at 2.60 mmol/L, and control the resting heart rate at about 60 beats per minute. And prohibit the application of other potassium channel openers. The course of medication is not limited.

#### 3.3.5 Exclusion criteria

(1) the content of the original literature is not about the clinical research of nicorandil in the treatment of microvascular angina, such as stable angina and coronary spasm.
(2) those who are allergic to nicorandil;
(3) chest pains caused by other causes, such as reflowal esoitis, aortic mezzanine, spontaneous gas chest, epidemic myalgia, rib cartilitis, myocarditis, acute thoracicitis, heart valve disease, rib fracture, shingles, vertical Tumours, pulmonary embolisms, cardiopulmonary encephalitis, and so on;
(4) severe liver and kidney dysfunction;
(5) abnormal thyroid function, severe electrolyte disorders;
(6) istemia of myocardial muscle caused by anemia and exovascular disease;
(7) cardiomyopathy, heart failure, heart-induced shock;
(8) other potassium ion openers that are not relevant to this study have been used.
(9) summary of the meeting, summary, case report and literature that failed to obtain the full text;
(10) documents that failed to obtain complete data and failed to obtain the original literature authors;
(11) animal studies.

### 3.4 Types of outcome

#### 3.4.1 Major outcome indicators

a. Seattle angina questionnaire;
b. Myocardial perfusion reserve index;
c. Coronary flow reserve;
d. Motion tablet observation, Duke Motion tablet observation is a validated indicator of dangerous stratified according to exercise time, ST segment low, and degree of angina while in motion: duke score = motion time (min) - 5 × ST segment decrease (mm) - (4 × angina index).

#### 3.4.2 Secondary outcomes.

1. The number of angina attacks;
2. The duration of angina;
3. Total number of ST segment depression leads;
4. Incidence of adverse reactions;
5. Level of endothelin-1;
6. Level of nitric oxide;
7. level of high-sensitivity C-reactive protein.

### 3.5 Research screening and data extraction

**Figure 1:**
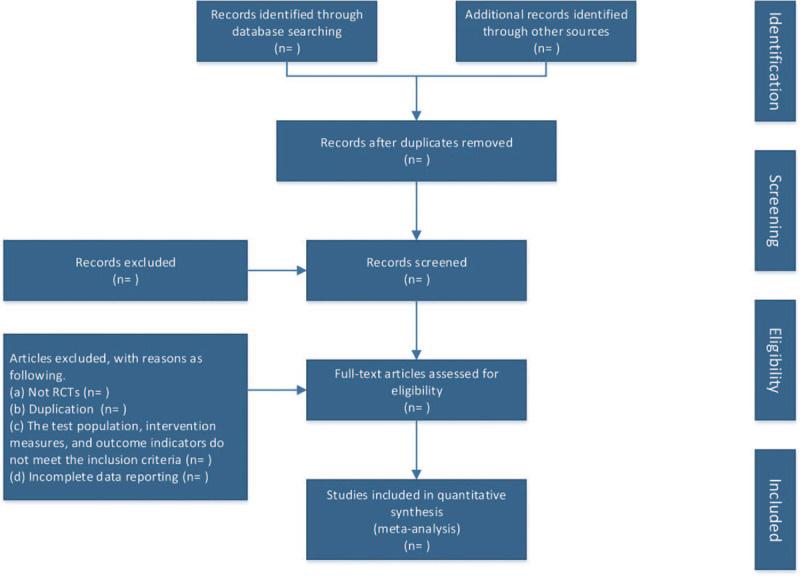
The whole process of research screening is shown by flow chart.

Two researchers independently screened the literature according to the inclusion and exclusion criteria mentioned above. If there are differences or other problems, seeking a third party to discuss and solve. Firstly, the collected literature is screened through the document management software EndNote X 9.3, and then the remaining literature is screened according to the inclusion and exclusion criteria. Finally, the relevant data of the included literature are sorted and summarized in the data extraction table. The flow chart is used to show the complete screening process of the literature (Fig. 1). The main contents of data extraction table include:

(1) Basic information, such as first author, journal name, publication time, research area, etc;
(2) The eligibility of the included literature, such as randomized control or not, whether the intervention measures meet the requirements, etc;
(3) The characteristics of the subjects, such as the total number of people, the measures and durations of the experimental group and the control group, the outcome indicators, and so on;
(4) Methodology, such as the generation of random scheme, concealment of allocation scheme, blind method, incomplete result data, selective report of results, and other bias, and so on;
(5) Outcome indicators, such as the type of outcome indicators (dichotomous variables, continuous variables, etc.).

### 3.6 Researches quality assessment

The studies included in this meta-analysis are randomized controlled trials, and the literature quality is evaluated according to the evaluation criteria of Cochrane Handbook, including the following 6 items: A. sequence generation; B. allocation concealment, equence generation and allocation concealment are both designed to prevent selection bias, which is caused by different selection conditions, and it may lead to overestimation or underestimation of the effect of intervention measures; C. blinding, in order to prevent the occurrence of implementation bias and measurement bias, the subjects and researchers do not know the grouping of trials, but the designer arranges and controls all trials. D. incomplete outcome data, incomplete result data will lead to follow-up bias, which refers to the exclusion of data due to withdrawal or loss of follow-up in the course of the trial or in the late follow-up period, the relevant data and reasons not stated in the literature or stated but not analyzed when included in the study; E. no selective outcome reporting will lead to reporting bias, which is caused by the existence of selective reporting results in the article; F. other sources of bias refer to other issues that may lead to a high risk of bias. Each bias is assessed according to 3 risk categories in Cochrane Handbook: unclear risk of bias, low risk of bias and high risk of bias.

### 3.7 Data synthesis

Using the Review Manager 5.3 software provided by the Cochrane Collaboration to conduct a meta-analysis on the final included literature.

#### 3.7.1 Consolidation of statistics

The research data included in the literature can be divided into hierarchical data and continuous variable data. According to different types of data, the combination methods of statistical data are also different.

(1) When the research data included in the literature is hierarchical data, it is necessary to convert the hierarchical data into binary variable data, and then select the more appropriate combination effect in the OR or RR for statistical analysis. OR is usually suitable for case-control studies; RR is suitable for cohort studies or randomized controlled trials.
(2) When the research data included in the literature is continuous variable data and the data units are consistent, the MD is selected for statistical analysis. If the data units are different, the SMD is selected for statistical analysis.

#### 3.7.2 Heterogeneity test

According to the statistical principle of meta-analysis, the data of different studies are heterogeneous. At this time, we need to test the heterogeneity of the included data. When the data of multiple studies have good homogeneity, the result index of heterogeneity test should show that *P* > .10, *I*^2^ is less than 50%, which indicates that the heterogeneity of multiple data is within the allowable range, so the fixed effect model should be used when merging data; if the heterogeneity of multiple research data exceeds the allowable range, the test result index is shown as *P* ≤ .10, *I*^2^≥ 50%, the source of heterogeneity can be found through subgroup analysis, or sensitivity analysis can be carried out to judge whether the results are accurate. If the test result index changes significantly and enters the allowable range during subgroup analysis or sensitivity analysis, the fixed effect model can be continued to be used for data consolidation, otherwise, If the result index of heterogeneity test does not change significantly and is still beyond the allowable range, the random effect model should be used to merge the data. Because the random effect model analysis has the characteristics of quantifying the bias among different studies, it can remove the heterogeneity caused by bias, and eliminate the impact of heterogeneity among different research data on the overall effect quantity, so that the estimated value of the effect quantity is closer to the real value.

#### 3.7.3 Sensitivity analysis

If there is obvious statistical heterogeneity between the included studies, the sensitivity analysis of the analysis results is needed.

(1) excluding studies of lower quality, excluding studies with less stringent designs, excluding studies with larger or small sample sizes, excluding studies that have not yet been published, and then re-examine meta-analysis to see whether the results change.
(2) after changing the study type and selecting different effect models for the same data, it is observed whether the effect merge value and confidence interval have changed.

If the meta-analysis results do not change significantly before and after the sensitivity analysis, which indicates that the meta-analysis results are reliable, and if the results of the analysis change significantly before and after the sensitivity analysis, which indicates that the reliability of the meta-analysis results is poor, the results and conclusions need to be described with caution.

#### 3.7.4 Subgroup analysis

According to the actual situation, this study will conduct a subgroup analysis of the heterogeneous outcome indicators in accordance with the use of nicorandil, the dosage, the treatment course of the drug, the age and gender of the patient to reduce the heterogeneity in the results and increase the stability of the results.

### 3.8 Publication Bias

For the analysis of potential publication bias, using funnel plots to show the outcome indicators of 7 or more trials. Funnel plots are scatter plots with the study effect as the horizontal axis and the sample size as the vertical axis. It is a method to identify and control publication bias in meta-analysis results. In theory, the point estimates of the research effects in meta-analysis should be distributed symmetrically and form an inverted and symmetrical “funnel” shape in the plane coordinate system. In general, the variation of effect quantity in small sample size research is large, and the effect quantity point estimation is usually scattered at the bottom of the plot; with the increase of sample size, the variation degree of effect quantity decreases, and the effect quantity point estimation is densely distributed in a narrow range in the upper part of the plot. If the plot is asymmetric or incomplete, it indicates that publication bias may exist. The severity of asymmetry or incompleteness of plot can reflect the size of publication bias.

### 3.9 Evidence evaluation

GRADE was evaluated by GRADE Profiler 3.6, which was analyzed from 5 aspects: limitation, inconsistency, indirectness, accuracy and publication bias. The level of evidence quality was expressed as “high,” “medium,” “low” and “very low.”

## 4 Discussion

MVA refers to patients with normal coronary angiography examination results, after excluding other organic heart diseases, the clinical manifestations are chest pain, myocardial ischemia and microcirculation disorders. In recent years, this disease has become a clinically frequent cardiovascular disease. With people's faster pace of life, the increased work pressure, the changes in diet and routines, the incidence of this disease has gradually increased, and it is becoming more and more threatening to people's life and health. At the same time, there are reports showing that the rate of MVA in female patients is greater than that in males.^[15]^ Since microvascular lesions are often accompanied by diseases such as hypertension and diabetes, the combination of this disease with other diseases is also increasing in clinical practice.^[16]^ The common causes of this disease are: the decrease in the number of circulating microvessels distributed on the myocardium, the dysfunction of vascular endothelial cells, the dysfunction of microvascular relaxation and contraction, etc. Its pathogenesis is the joint participation of various pathophysiological changes. The regulation of coronary blood flow relies on the normal diastolic and contraction of coronary microcirculation blood vessels, which in turn depends on the normal function of microvascular endothelial cells.^[17,18]^ As we all know, vascular endothelial cells can synthesize and release substances that constrict blood vessels as well as substances that relax blood vessels. The former has a drastic vasoconstriction effect, such as endothelin-1 and angiotensin-II; the latter such as NO and prostacyclin can play a role in vasodilation.^[19]^ Chauhan A considered that the possible multi-faceted pathogenesis of MVA patients may be due to the following reasons: the reduction of NO synthesis and release, which depends on the impaired vasodilation function of endothelial cells, and there may also be NO that can no longer antagonize the contraction of blood vessels.^[20]^ This adverse reaction often occurs in the case of excessive release of endothelin. Patients with poor microvascular vasodilation, decreased coronary flow, insufficient myocardial blood supply, and imbalance of oxygen supply and demand, leading to angina pectoris and changes in the ischemic ST segment of electrocardiogram. In addition, atherosclerosis, intercellular adhesion molecules, abnormal blood lipids or hemorheology, hyperhomocysteinemia, insulin resistance or diabetes, low or lack of estrogen, high blood pressure, smoking and many other factors can also cause coronary disease. Vascular microvascular endothelial cells are dysfunctional, and they are often accompanied by the participation of inflammatory mechanisms.

Nowadays, western medicine is still based on the standardized treatment of stable angina to deal with microvascular angina. The first is to control the risk factors of atherosclerosis, and the second is to reduce the myocardial oxygen consumption of patients. In microvascular angina, the first-line drugs are β blockers, calcium channel blockers (non-dihydropyridines), and ATP sensitive potassium channel openers, such as nicorandil.^[21,22]^ In the past randomized controlled clinical trials, nicorandil can improve the symptoms of angina pectoris, electrocardiogram and motion tablet test results have also improved to a certain extent. Nicorandil is an anti-angina drug called n - (2-hydroxyethyl) nicotinamide nitrate, which has more than ATP. The sensitive potassium channel opener has antiarrhythmic effect. At the same time, because of the nitrate group in its molecular structure, it also has the effect of nitrates to dilate blood vessels, improve the coronary blood flow and reduce the preload and afterload of the heart. Nicorandil can effectively treat unstable angina and its related microvascular complications. Nicorandil has been shown to improve microvascular perfusion, relieve microvascular spasm and reduce platelet aggregation in animal and clinical trials. The mechanism of myocardial protection may be related to the opening of ATP-sensitive potassium channel, reducing the production of excessive oxygen free radicals during myocardial ischemia, improving the antioxidant capacity of myocardium, and inhibiting the apoptosis and inflammatory reaction of myocardial cells after ischemia.^[23]^

Nicorandil is currently a more in-depth clinically researched drug. Because of its special dual effects, nicorandil has been used in many aspects of the treatment of heart diseases. Its oral preparations can be used to treat arrhythmias, chronic heart failure, stable angina, and acute coronary syndromes (including post-PCI). At this stage, nicorandil has been used in the treatment of microvascular angina, but there is a lack of high-quality randomized controlled trials on the clinical effectiveness and safety of nicorandil in the treatment of microvascular angina, and there is a lack of evaluation of its effectiveness and safety. Therefore, a positive conclusion will be given through this research conference, and the relevant research results of this research will provide a theoretical basis for the drug treatment of microvascular angina and benefit more cardiovascular disease patients.

## Author contributions

**Conceptualization:** Conghong Shi, Ye Zhang, Xiaojuan Wang.

**Data curation:** Ye Zhang, Xiaojuan Wang, Ruijuan Liu, Qingsheng Li, Wei Tian, Hong Lei.

**Formal analysis:** Ye Zhang, Xiaojuan Wang, Ruijuan Liu, Qingsheng Li.

**Funding acquisition:** Ye Zhang.

**Resources:** Ye Zhang, Xiaojuan Wang, Ruijuan Liu, Hong Lei.

**Software:** Ye Zhang, Xiaojuan Wang, Qingsheng Li, Wei Tian.

**Supervision:** Conghong Shi, Ye Zhang, Xiaojuan Wang

**Writing – original draft:** Ye Zhang, Xiaojuan Wang, Ruijuan Liu, Qingsheng Li, Wei Tian, Hong Lei.

**Writing – review & editing:** Conghong Shi.

## Abbreviations

Abbreviations: ATP = adenosine triphosphate, MVA = microvascular angina, NO = nitric oxide.
